# One-year outcomes after treatment with a drug-coated balloon catheter system for lower urinary tract symptoms related to benign prostatic hyperplasia

**DOI:** 10.1038/s41391-021-00362-z

**Published:** 2021-04-08

**Authors:** Steven A. Kaplan, Merycarla Pichardo, Edwin Rijo, Gustavo Espino, Ramon Rodriguez Lay, Rafael Estrella

**Affiliations:** 1 Department of Urology, Icahn School of Medicine at Mount Sinai, New York City, NY USA; 2 URUS, Santo Domingo, Dominican Republic; 3 Centro Médico Dr. Canela SRL, La Romana, Dominican Republic; 4 Centro Especializado San Fernando, Panama City, Panama; 5 Edificio Royal Center, Panama City, Panama; 6 Clínica Unión Medica, Santiago de los Caballeros, Dominican Republic

**Keywords:** Prostatic diseases, Outcomes research

## Abstract

**Background:**

This is the first report of the 1-year outcomes of the EVEREST-I study evaluating the safety and efficacy of the Optilume^®^ BPH Catheter System, a prostatic paclitaxel-coated balloon catheter system, for the treatment of lower urinary tract symptoms (LUTS) related to benign prostatic hyperplasia (BPH).

**Methods:**

Subjects were men >50 years old with moderate-to-severe LUTS secondary to BPH, peak urinary flow rate of 5–15 ml/s, prostatic urethra length 30–55 mm, and prostate volume 20–80 g. All were treated with the Optilume BPH Catheter System and followed at Foley removal, 2 weeks, 30 days, 3, 6, and 12 months after treatment. The primary endpoint was the proportion of subjects with ≥40% improvement in International Prostate Symptom Score (IPSS). The rate of post-procedural complications was evaluated.

**Results:**

Eighty subjects were treated at six sites in Latin America and 75 completed the 1-year follow-up. The percent of subjects with an improvement ≥40% in IPSS from baseline was 81% at 3 months and 1 year. IPSS improved from 22.3 at baseline to 7.9 at 1 year, Qmax improved from 10.9 to 18.4 ml/s, and IPSS QoL improved from 4.6 to 1.3. Post-procedural complications included common urologic events and the rate of complications was significantly impacted by device diameter.

**Conclusions:**

Treatment with the minimally invasive Optilume BPH Catheter System is safe and showed subjective and objective improvements in LUTS. Benefits were rapid and persisted through 1 year. The initial results warrant further evaluation of this therapy as a treatment option for patients with LUTS related to BPH.

## Introduction

Benign prostatic hyperplasia (BPH) with associated lower urinary tract symptoms (LUTS) is a common medical condition in the aging male. The prevalence is estimated at 20% for men in their 40s increasing to 80–90% for men in their 70s and 80s [1]. Symptoms of bladder outlet obstruction and bladder irritability can significantly impact patient quality of life (QoL).

Transurethral resection of the prostate, whether by electrosurgery or laser, is considered the gold standard for the treatment of moderate-to-severe LUTS/BPH. However, it is an invasive option and is associated with long-term complications including retrograde ejaculation and erectile dysfunction [2]. As a result, minimally invasive therapies have been developed that aim to achieve similar efficacy with reduced side effects. These therapies include permanent implants that lift and hold open the prostatic urethra (e.g., prostatic urethral lift [PUL]) and devices that use various forms of energy to resect or ablate tissue to relieve the bladder obstruction [3]. There is a continued clinical need to fill the gap between medication and surgical therapies for treatments that provide significant and durable symptom relief, while minimizing morbidity and improving patient QoL.

**Fig. 1:**
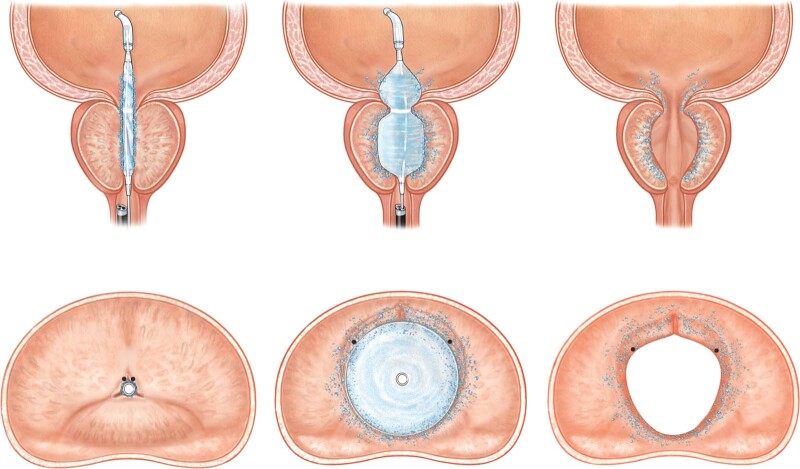
Positioning and inflation of the Optilume BPH DCB from sagittal (top) and transverse (bottom) planes. Inflation of the balloon leads to a splitting of the anterior commissure with concurrent delivery of paclitaxel to the prostatic urethra.

Clinical studies on transurethral dilation of the prostate conducted in the 1990s showed that balloon dilation techniques were safe; however, long-term durability was a problem due to healing of the commissure, scar tissue formation, or general recovery of the initially compressed tissue [4, 5]. The Optilume^®^ BPH Catheter System (Urotronic Inc., Plymouth, Minnesota, USA) is the first prostatic drug-coated balloon dilation system for the treatment of obstructive BPH. The system combines mechanical dilation to achieve commissurotomy of the anterior prostatic urethra to open the urethral lumen, with the delivery of the drug paclitaxel to the dilated area to maintain urethral patency (Fig. 1). Paclitaxel is an antiproliferative drug that has been extensively used in vascular devices to prevent restenosis after balloon angioplasty or stenting [6]. The goal of this technology is to provide immediate symptomatic relief via balloon dilation and to achieve durable results via localized paclitaxel delivery. We report the 1-year outcomes of the first clinical experience with the Optilume BPH Catheter System in men with moderate-to-severe LUTS secondary to BPH.

## Materials and methods

### Study design

EVEREST-I is a prospective, single-arm, non-randomized, open label, multicenter study conducted at six sites in Latin America (clinicaltrials.gov: NCT03423979). Ethics Committee approval was obtained for all study sites prior to study commencement.

The study was designed to assess the safety and efficacy of the Optilume BPH Catheter System for the treatment of LUTS secondary to BPH. Subjects were followed at 2–5 days (Foley removal), 2 weeks, 30 days, 3 months, 6 months, and 1-year post treatment for outcome assessments. Some subjects participated in a pharmacokinetics study to evaluate drug content in plasma, urine, and semen.

The primary efficacy endpoint was the responder rate at 3 months based on the improvement from baseline in International Prostate Symptom Score (IPSS). A responder was defined as a subject with IPSS improvement ≥40% from baseline without requiring additional therapy. Secondary endpoint assessments included IPSS, peak urinary flow rate (Qmax), and responder rate based on varying IPSS improvement thresholds. Pain was assessed using the visual analog scale (VAS) at baseline, immediately post-procedure, Foley removal, 2 weeks, and 30 days. Sexual function was assessed using the International Index of Erectile Function (IIEF) and Male Sexual Health Questionnaire-Ejaculatory Dysfunction (MSHQ-EjD) questionnaires. Adverse events (AEs) were reported starting from the time the procedure was conducted. All events were adjudicated by the study principal investigator.

### Study subjects

Eligible subjects were males >50 years of age with LUTS secondary to BPH, with IPSS ≥ 13, Qmax 5–15 ml/s, post-void residual (PVR) volume ≤250 ml, prostate volume 20–80 g, and prostatic urethra length 35–55 mm. Key protocol exclusions included prior minimally invasive or surgical intervention of the prostate, intravesical prostatic protrusion >1 cm, and confounding urologic conditions (e.g., neurogenic bladder, stricture). Subjects had to undergo drug washouts prior to treatment including alpha blockers for 3 weeks and 5-alpha reductase inhibitors for 6 months. Written informed consent was obtained from all subjects.

### Study device and procedure

The Optilume BPH Catheter System is comprised of two balloon catheters; a pre-dilation balloon to initiate the anterior commissurotomy and a drug-coated balloon to further dilate and transfer paclitaxel to the prostatic urethra. The proprietary balloon design “locks” the balloon in place during inflation and ensures that the dilating force is applied directly to the prostatic fossa, sparing the bladder neck and limiting slippage of the balloon into the bladder. The paclitaxel acts to limit growth of the prostatic adenoma and scar tissue formation within the anterior commissurotomy in the postoperative period while allowing the urothelium to regenerate. The expected result is an immediate increase in cross-sectional area of the prostatic urethra from the anterior commissurotomy, which is maintained by limiting scar tissue and adenoma growth in the short term and by establishing full coverage of the urothelium within the commissurotomy to prevent lateral lobe fusion in the long term.

The study procedure was conducted under general anesthesia or spinal block in accordance with local standards. Balloon sizing was determined based on transrectal ultrasound measurements of the prostate volume and length prior to initiating the procedure. Balloon diameter and length were selected in accordance with an established sizing grid. To begin the procedure, the pre-dilation balloon was advanced into the urethra, positioned with the proximal balloon tail at the level of the external sphincter, and inflated to initiate the anterior commissurotomy. After a short inflation, the pre-dilation balloon was withdrawn, and the drug-coated balloon was then advanced in a similar manner and inflated for a minimum of 5 min to complete the anterior commissurotomy and deliver the paclitaxel to the prostatic urethral surface. After completion of the dilation procedure, a Foley catheter was placed for a minimum of 48 h.

### Statistical methods

The primary efficacy analysis was based on a modified intent-to-treat population that included all ITT subjects who had the investigational device attempted. For the primary endpoint, subjects who exited the study for any reason prior to the 3-month visit were assumed non-responders. A 90% two-sided confidence interval (CI) for the responder rate was estimated using the exact Clopper–Pearson interval. The primary endpoint was met if the lower bound of the 90% CI was greater than the responder rate performance goal of 50%. For other assessments, appropriate summary statistics were used. A sample size of 42 subjects was needed to provide 80% power with a 0.05 one-sided type I error to meet the responder rate performance goal of 50%, assuming an expected responder rate at 3 months of 70%.

## Results

### Study population and treatment procedure

A total of 80 male subjects enrolled in the study at two sites in Panama and four sites in the Dominican Republic. Subjects averaged 65.8 ± 7.8 years of age and had a prostate volume of 35.9 ± 13.2 g. The majority (85.0%) were of Hispanic or Latino ethnicity.

All enrolled subjects were treated with the Optilume BPH Catheter System. Pain intensity post procedure was low, with average VAS of 0.8 ± 1.5 at baseline, 1.7 ± 2.3 immediately post procedure, 1.8 ± 2.1 at Foley removal, and 1.0 ± 2.0 by 30 days. Of the 80 subjects treated, 5 did not complete the 1-year visit.

### Efficacy

The primary efficacy endpoint was met (Table 1). Of the 80 subjects treated, 1 died of non-study related causes prior to the 3-month visit and was considered a non-responder. The percentage of subjects with ≥40% reduction in IPSS from baseline was 81.3% (65/80) at 3 months with a 90% lower confidence bound of 72.6% that exceeded the pre-specified performance goal of 50%.

**Table 1:** Responder rate (≥40% IPSS improvement) at 3 months.

Analysis	Responder rate, % (*n*/*N*)	90% CI
Primary efficacy analysis, worst case	81.3 (65/80)	(72.6, 88.1)
Additional analysis, complete case	82.3 (65/79)	(73.7, 89.0)

**Fig. 2:**
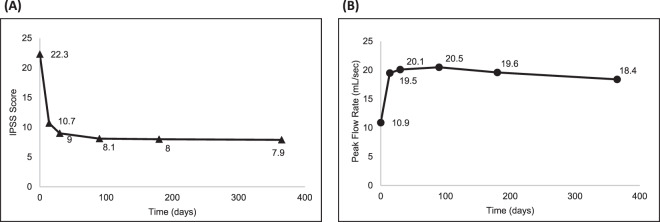
Improvement in symptom severity and peak urinary flow rate. Mean improvement in **A** IPSS and **B** Qmax through 1 year.

Secondary outcome measures showed sustained improvement through 1 year of post-procedural follow-up (Table 2 and Fig. 2). The average IPSS decreased from 22.3 at baseline to 8.1 at 3 months, 8.0 at 6 months, and 7.9 at 1 year. This corresponds to an average improvement of 63.0%, 64.0%, and 64.9%, respectively. More than 70% of subjects had at least a 50% improvement in IPSS at each of the follow-up visits beginning at 30 days after the treatment procedure. Approximately half of the subjects (50.7%) experienced a 75% improvement in IPSS by 1 year. Voiding function as assessed by peak urinary flow also improved after treatment. The average Qmax increased from 10.9 ml/s at baseline to 20.5 ml/s at 3 months, 19.6 ml/s at 6 months, and 18.4 ml/s at 1 year, corresponding to an improvement of 9.6, 8.8, and 7.4 ml/s, respectively. Improvements in IPSS and uroflowmetry measures were accompanied by an improvement in QoL. The average IPSS QoL score decreased from 4.6 at baseline to 1.3 at 1 year, representing an improvement of 70.7%.

**Table 2:** Secondary outcomes summary.

Measure	Baseline	2 weeks	30 days	3 months	6 months	1 year
*IPSS*						
*n*	80	78	79	79	77	75
Mean ± SD	22.3 ± 4.9	10.7 ± 6.4	9.0 ± 6.2	8.1 ± 6.1	8.0 ± 7.2	7.9 ± 7.6
Mean change	NA	−11.7	−13.4	−14.2	−14.4	−14.4
% Change	NA	−51.5	−59.1	−63.0	−64.0	−64.9
*IPSS QoL*						
*n*	80	78	79	79	77	75
Mean ± SD	4.6 ± 0.9	1.8 ± 1.5	1.6 ± 1.4	1.5 ± 1.3	1.6 ± 1.6	1.3 ± 1.4
Mean change	NA	−2.9	−3.0	−3.1	−3.0	−3.3
% Change	NA	−62.0	−63.9	−67.1	−61.1	−70.7
*Qmax (ml/s)*						
*n*	80	72	79	77	74	74
Mean ± SD	10.9 ± 2.9	19.5 ± 10.2	20.1 ± 9.4	20.5 ± 9.5	19.6 ± 8.7	18.4 ± 8.2
Mean change	NA	8.5	9.2	9.6	8.8	7.4
*PVR (ml)*						
*n*	80	77	79	78	75	75
Mean ± SD	63.1 ± 55.0	44.0 ± 56.8	29.9 ± 31.8	33.9 ± 33.1	29.7 ± 30.5	33.9 ± 35.2
Mean change	NA	−20.4	−33.4	−29.7	−34.6	−29.8
*Responder rate*						
≥30%						
*n*/*N* (%)	NA	64/78 (82.1)	68/79 (86.1)	71/79 (89.9)	66/77 (85.7)	65/75 (86.7)
95% CI		71.7, 89.8	76.5, 92.8	81.0, 95.5	75.9, 92.6	76.8, 93.4
≥40%						
*n*/*N* (%)	NA	58/78 (74.4)	65/79 (82.3)	65/79 (82.3)	61/77 (79.2)	61/75 (81.3)
95% CI		63.2, 83.6	72.1, 90.0	72.1, 90.0	68.5, 87.6	70.7, 89.4
≥50%						
*n*/*N* (%)	NA	50/78 (64.1)	58/79 (73.4)	57/79 (72.2)	54/77 (70.1)	55/75 (73.3)
95% CI		52.4, 74.7	62.3, 82.7	60.9, 81.7	58.6, 80.0	61.9, 82.9
≥75%						
*n*/*N* (%)	NA	14/78 (17.9)	25/79 (31.6)	36/79 (45.6)	36/77 (46.8)	38/75 (50.7)
95% CI		10.2, 28.3	21.6, 43.1	34.3, 57.2	35.3, 58.5	38.9, 62.4

There was no deterioration in sexual function based on patient reported assessments (Table 3). Erectile function as measured by the overall satisfaction domain of the IIEF was stable over time, with a slight improvement from baseline at 1 year. The average IIEF score was 7.2 at baseline and 7.9 at 1 year. Similarly, ejaculatory function as measured by MSHQ-EjD was also preserved. The ejaculatory function score was 9.4 at baseline and 9.1 at 1 year, while the average bother score was 1.2 at 1 year remaining unchanged from baseline.

**Table 3:** IIEF overall satisfaction and MSHQ-EjD function and bother.

Measure	Baseline	30 days	3 months	6 months	1 year
*IIEF overall satisfaction*					
*n*	80	79	79	76	75
Mean ± SD	7.2 ± 2.4	7.4 ± 2.0	7.2 ± 2.4	7.8 ± 2.1	7.9 ± 2.1
*MSHQ-EjD function*					
*n*	80	79	79	77	75
Mean ± SD	9.4 ± 4.3	7.5 ± 5.8	8.7 ± 5.6	8.1 ± 5.8	9.1 ± 5.7
*MSHQ-EjD bother*					
*n*	80	79	79	77	75
Mean ± SD	1.2 ± 1.4	1.3 ± 1.6	1.2 ± 1.5	1.3 ± 1.6	1.2 ± 1.5

### Safety

A total of 113 AEs were reported through 1 year with most (84/113) occurring within 3 months after the procedure. The most frequent treatment-related AEs were post-procedural hematuria (15.0%), postoperative urinary retention (13.8%), urinary incontinence (13.8%), urinary tract infection (8.8%), ejaculation disorder (8.8%), and dysuria (7.5%). Postoperative urinary retention events were largely due to clots blocking the Foley catheter outlet with >90% (11/12) of events resolving within 1 week. Bleeding-related AEs occurred at a rate of 28.8% (23/80) estimated by pooling events of hematuria and postoperative urinary retention (events due to clots). There were no unanticipated adverse device-related events. Two deaths unrelated to treatment were reported, one due to an underlying pre-existing medical condition resulting in a stroke and one due to a bowel obstruction.

An interim analysis of the data found a relatively poorer safety profile when using the large diameter balloon catheter, including higher rates of bleeding and incontinence-related events, leading to removal of this device size option for the last 31 subjects in the study. All four stress incontinence events in the study were associated with use of the large diameter balloon catheter. Bleeding-related rates were 54.5% (12/22) and 19.0% (11/58) for subjects treated with large and small/medium DCBs, respectively. A similar trend was found for treatment-related incontinence events with a rate of 45.5% (10/22) for large DCBs and 1.7% (1/58) for smaller DCBs. The single incontinence event in the small/medium diameter group was urge incontinence secondary to overactive bladder.

As expected, the urine paclitaxel concentration was highest immediately after the procedure at 598 ± 475 ng/ml (*n* = 45) that decreased to 203 ± 444 ng/ml (*n* = 41) at 4 days, and 5 ± 11 ng/ml (*n* = 42) at 2 weeks. Paclitaxel concentrations in plasma and semen were very low and close to the limit of quantification post procedure. The average plasma paclitaxel concentration was 0.25 ± 0.38 ng/ml (*n* = 31) immediately after the procedure and decreased to below the level of quantification by 10 h (*n* = 30). The average semen paclitaxel concentration was 13 ± 25 ng/ml (*n* = 19) at 2 weeks that decreased to 0.1 ± 0.2 ng/ml (*n* = 14) at 6 months.

## Discussion

The results of the EVEREST-I study showed that treatment with the Optilume BPH Catheter System was safe and could achieve a rapid and sustained reduction in the severity of LUTS through 1 year. The primary efficacy analysis showed that 81.3% of subjects experienced ≥40% improvement in IPSS at 3 months. Results were consistent across all analyses of the primary endpoint.

Subjects enrolled in the study had severe symptoms at baseline with an average IPSS of 22.3 that decreased to 10.7 at 2 weeks and 7.9 at 1 year. The reduction in IPSS of 14.4 points at 1 year represents a clinically meaningful improvement based on a minimum clinically important difference of 6 points reported for patients with severe LUTS [7]. Compared to published data of other minimally invasive therapies, although in different patient populations, the Optilume BPH Catheter System showed improved and more rapid symptom relief. Baseline, 2-week, and 1-year scores for UroLift PUL were 22.2, 18.0, and 11.1, respectively [8]. Corresponding values for the Rezūm water vapor thermal therapy were 22.0, 18.6, and 10.2 [9].

Subjective improvements in IPSS were accompanied by objective improvements in uroflowmetry as evidenced by Qmax and PVR volume results. Peak urinary flow increased from 10.9 ml/s at baseline to 18.4 ml/s at 1 year. As with IPSS, these represent clinically meaningful improvements with an increase of 2 ml/s considered the minimum clinically important difference [10]. The improvement in Qmax compares favorably to published data for UroLift and Rezūm devices. Average baseline and 1-year values in the UroLift study were 8.9 and 12.1 ml/s, respectively, while corresponding values for the Rezūm study were 9.9 and 14.9 ml/s.

Subjects experienced an average improvement of 70.7% in QoL, potentially driven by the reduction in symptom severity but also by the maintenance of sexual function [11]. Based on the IIEF and MSHQ-EjD results, treatment with the device had no effect on perceived sexual function through 1 year.

Most events reported in the study were common post-urinary intervention AEs, such as urinary tract infection, urinary retention, or incontinence. The types and rates of treatment-related AEs were similar to those reported in trials evaluating other minimally invasive therapies for symptomatic BPH [8, 9]. The rate of treatment-related bleeding AEs for the Optilume BPH Catheter System was 28.8% (23/80), which is similar to the rate reported for UroLift of 26.4% (37/140) for hematuria; the rate is lower for Optilume BPH if excluding the large device diameter (19.0%, 11/58). Both bleeding and incontinence complication rates were greatly reduced when excluding events occurring with large diameter balloon catheters, with the safety profile of the smaller diameter balloon catheters being reflective of the current therapy and the ongoing pivotal PINNACLE trial.

Pharmacokinetic data supported the safety of the drug coating with very low levels of paclitaxel found in plasma and semen indicating that the risk of systemic drug toxicity is low. As expected, the only significant paclitaxel concentration was found immediately post procedure in urine that decreased thereafter, suggesting that the drug is localized to the treatment area of the urethra and is removed from the body during voiding.

Study limitations include the lack of a control group. A randomized clinical trial is currently ongoing to confirm the findings. This initial experience helped inform development of the technology to improve outcomes. In this case, removal of the large diameter balloon led to an improved safety profile with no impact to efficacy as compared to the overall cohort. When considering the smaller diameter balloon catheter subgroup, 86.2% (50/58) achieved a 40% reduction in IPSS at 3 months and none of these subjects experienced a major treatment-related complication. The average IPSS was reduced from 22.2 at baseline to 7.8 at 1 year in this subgroup. Longer term follow-up is needed to collect robust durability data for the device.

## Conclusion

Clinical data from the EVEREST-I study showed that treatment for obstructive BPH with Optilume BPH Catheter System provides clinically meaningful benefits, with rapid improvement in LUTS severity and voiding function within 2 weeks that were sustained through 1 year. The therapy had an acceptable safety profile and improved subject’s QoL. This new technological approach shows promise as an effective minimally invasive treatment alternative to medication or surgical interventions without the necessity of a permanent implant or tissue ablation/removal.
