# Correction: One-year outcomes after treatment with a drug-coated balloon catheter system for lower urinary tract symptoms related to benign prostatic hyperplasia

**DOI:** 10.1038/s41391-021-00414-4

**Published:** 2021-07-07

**Authors:** Steven A. Kaplan, Merycarla Pichardo, Edwin Rijo, Gustavo Espino, Ramon Rodriguez Lay, Rafael Estrella

**Affiliations:** 1 Department of Urology, Icahn School of Medicine at Mount Sinai, New York, NY USA; 2 URUS, Santo Domingo, Dominican Republic; 3 Centro Médico Dr. Canela SRL, La Romana, Dominican Republic; 4 Centro Especializado San Fernando, Panama City, Panama; 5 Edificio Royal Center, Panama City, Panama; 6 Clínica Unión Medica, Santiago de los Caballeros, Dominican Republic

**Keywords:** Prostatic diseases, Outcomes research

Correction to: Prostate Cancer and Prostatic Diseases 10.1038/s41391-021-00362-z

The article “One-year outcomes after treatment with a drug-coated balloon catheter system for lower urinary tract symptoms related to benign prostatic hyperplasia”, written by Steven A. Kaplan, Merycarla Pichardo, Edwin Rijo, Gustavo Espino, Ramon Rodriguez Lay, and Rafael Estrella was originally published online on 23 March 2021 without open access. With the author(s)’ decision to opt for Open Choice the copyright of the article changed on 20th May 2021 to © The Author(s) 2021 and the article is forthwith distributed under a Creative Commons Attribution 4.0 International License, which permits use, sharing, adaptation, distribution, and reproduction in any medium or format, as long as you give appropriate credit to the original author(s) and the source, provide a link to the Creative Commons license, and indicate if changes were made. The images or other third-party material in this article are included in the article’s Creative Commons license unless indicated otherwise in a credit line to the material. If material is not included in the article’s Creative Commons license and your intended use is not permitted by statutory regulation or exceeds the permitted use, you will need to obtain permission directly from the copyright holder. To view a copy of this license, visit http://creativecommons.org/licenses/by/4.0/.

